# *CYP2D6* Genotype Predicts Plasma Concentrations of Tamoxifen Metabolites in Ethiopian Breast Cancer Patients

**DOI:** 10.3390/cancers11091353

**Published:** 2019-09-12

**Authors:** Jemal Hussien Ahmed, Eyasu Makonnen, Alan Fotoohi, Abraham Aseffa, Rawleigh Howe, Eleni Aklillu

**Affiliations:** 1 Department of Pharmacology and Clinical Pharmacy, Addis Ababa University, Addis Ababa P.O. Box 9086, Ethiopiaeyasumakonnen@yahoo.com (E.M.); 2 Division of Clinical Pharmacology, Department of Laboratory Medicine, Karolinska Institutet, Karolinska University Hospital, Huddinge, Stockholm 141 86, Sweden; 3 Center for Innovative Drug Development and Therapeutic Trials, Addis Ababa University, Addis Ababa P.O. Box 9086, Ethiopia; 4 Division of Clinical Pharmacology, Department of Medicine, Karolinska Institutet, Solna Stockholm 171 76, Sweden; Alan.Fotoohi@ki.se; 5 Armauer Hansen Research Institute, Addis Ababa P.O. Box 1005, Ethiopia; aseffaa@gmail.com (A.A.); rawcraig@yahoo.com (R.H.)

**Keywords:** tamoxifen, endoxifen, pharmacokinetics, pharmacogenetics, *CYP2D6*, *ABCB1*, breast cancer

## Abstract

Tamoxifen displays wide inter-individual variability (IIV) in its pharmacokinetics and treatment outcome. Data on tamoxifen pharmacokinetics and pharmacogenetics from black African breast cancer patient populations is lacking. We investigated the pharmacokinetic and pharmacogenetic profile of tamoxifen and its major active metabolite, endoxifen, in Ethiopian breast cancer patients. A total of 81 female breast cancer patients on adjuvant tamoxifen therapy were enrolled. Tamoxifen (Tam) and its major metabolites, N-desmethyltamoxifen (NDM), 4-hydroxy-tamoxifen (4-HT), and (*Z*)-endoxifen (E) were quantified using LC-MS/MS. Genotyping for *CYP2D6, CYP2C9, CYP2C19, CYP3A5, POR,* and *ABCB1* and *UGT2B1*5 and copy number variation for *CYP2D6* were done. The proportion of patients with low endoxifen level (<5.9 ng/mL) was 35.8% (median concentration 7.94 ng/mL). The allele frequency of *CYP2D6* gene deletion (*5) and duplication (*1×N or *2×N) was 4.3% and 14.8%, respectively. Twenty-six percent of the patients carried duplicated or multiplicated *CYP2D6* gene. An increase in *CYP2D6* activity score was associated with increased endoxifen concentration and MR_E/NDM_ (*p* < 0.001). The IIV in endoxifen concentration and MR_E/NDM_ was 74.6% and 59%, respectively. *CYP2D6* diplotype explained 28.2% and 44% of the variability in absolute endoxifen concentration and MR_E/NDM_, respectively. The explanatory power of *CYP2D6* diplotype was improved among *ABCB1c*.*4036G* carriers (43% and 65.2%, respectively for endoxifen concentration and MR_E/NDM_) compared to *A/A* genotype. *CYP2C9*, *CYP2C19*, and *CYP3A5* genotypes had no significant influence on endoxifen concentration or MR_E/NDM._ In conclusion, we report a high rate of low endoxifen level as well as large IIV in tamoxifen and its metabolite concentrations. *CYP2D6* is significant predictor of plasma endoxifen level in a gene-dose dependent manner.

## 1. Introduction

Tamoxifen is widely used as adjuvant therapy for women with estrogen receptor-positive breast cancer [1]. Its use for 5 years has been shown to be highly effective in reducing breast cancer recurrence and cancer-specific mortality [2]. Despite its proven clinical effectiveness, not all breast cancer patients gain benefit from tamoxifen treatment; approximately 30–50% of patients relapse or eventually die from the disease [2]. Some individuals also experience treatment associated side effects [3].

The causes for breast cancer relapse or occurrence of side effects after tamoxifen treatment are attributed to a variety of factors, including pharmacogenetic variations in relevant drug-metabolizing enzymes involved in the metabolic activation of tamoxifen into endoxifen [1,4]. The bioactivation of tamoxifen involves several CYP450 drug metabolizing enzymes (DMEs) including, *CYP2D6, CYP3A4, CYP3A5, CYP2C9,* and *CYP2C19*, with key roles for *CYP2D6* and *CYP3A* [5]. The wide interindividual variability in the concentration of tamoxifen and its metabolites was mainly attributed to genetic polymorphism in *CYP2D6* [3,6,7,8]. Previous studies showed that patients with low *CYP2D6* enzyme activity or taking medication which inhibit *CYP2D6* activity were associated with lower endoxifen concentration [7,9]. Other studies also demonstrated that patients exhibiting endoxifen concentration below a proposed therapeutic threshold (5.9 ng/mL), were significantly associated with a higher risk of recurrence or death [3,10].

The link between patients’ *CYP2D6* genotype and plasma levels of tamoxifen and its metabolites has been established in population of European and Asian descent [10,11,12], but not in sub-Saharan populations. Studies also showed that *CYP2D6* explains about 39–58% of the interpatient variability in endoxifen concentration [8] and residual variability is largely unexplained [7,12]. The extent to which *CYP2D6* determines the wide interpatient variability of endoxifen under standard tamoxifen treatment is not well characterized in black African populations. Although *CYP2D6* is the predominant enzyme in the metabolic conversion of tamoxifen into endoxifen, previous studies reported inconsistent findings about the association between *CYP2D6* polymorphism and clinical outcome of tamoxifen therapy.

The polymorphic *CYP2D6* gene exhibits pronounced interethnic differences in allele frequency distribution. Ethnic differences in tamoxifen metabolism and treatment outcome are also well recognized [13,14]. Reports on the association of *CYP2D6* genotype with clinical outcome in different population is not consistent, and hence lack of consensus to recommend genotype-based personalized tamoxifen treatment. About 29% of Ethiopians carry functionally active *CYP2D6* gene duplications and multiplications associated with increased enzyme activity [15]. Given this unique *CYP2D6* genotype constitution, the pharmacokinetic profile of tamoxifen in this population may have important implications for tamoxifen dosing recommendations to improve clinical effectiveness of the treatment. *CYP2C9*, *CYP2C19*, and *CYP3A5* are also involved in tamoxifen metabolism, but the impact of their genotype on the pharmacokinetics (PK) of tamoxifen are conflicting [4,6]. Furthermore, data on the impact of other genetic variations such as the role of *POR* and non-genetic determinants on endoxifen plasma concentration is limited. There is growing evidence that altered *ABCB1* activity may affect tamoxifen pharmacokinetics and possibly tamoxifen efficacy in breast cancer patients. The primary active tamoxifen metabolites, endoxifen and 4-hydroxytamoxifen, are substrates for *ABCB1* [16]. The three common SNPs in the protein coding region of *ABCB1* gene (rs1128503 (1236T>C), rs2032582 (2677T>G/A), and rs1045642 (3435T>C) have been the focus of many pharmacokinetic and disease association studies [17] with inconsistent and controversial results [18]. Other studies have also investigated the relationship between the C3435T polymorphism of *ABCB1* gene and risk of breast cancer; whose results are also conflicting [19,20]. On the other hand, the relevance of *ABCB1*c.4036A>G (rs3842A>G) has, however, been consistently demonstrated in African population in relation to the PK of antiretroviral drugs. Several studies have showed that *ABCB1*c.4036A>G (rs3842A>G) is a significant predictor of plasma and intracellular efavirenz concentration in Uganda [21,22], South Africa [23], Ethiopia and Tanzania [24] as well as in other populations [25]. We hypothesize that this SNP may also influence cellular transport of tamoxifen metabolites in our study population. In the present study, we investigated the pharmacokinetics profile and the influence of *CYP2D6*, *CYP2C9*, *CYP2C19*, *CYP3A5*, *POR, ABCB1* and *UGT2B1*5 polymorphisms on the plasma level of tamoxifen and its active metabolites in Ethiopian breast cancer patients receiving adjuvant tamoxifen.

## 2. Results

### 2.1. Sociodemographic Characterstics

A total of 89 patients (mean age = 39 ± 8.5) who were on tamoxifen treatment were enrolled. Of these, eight patients were excluded as the plasma level of tamoxifen and endoxifen were below the limit of quantification, possibly related to noncompliance to therapy. The remaining 81 patients, all with tamoxifen concentration >150 nM were included in the analysis. All the patients completed full course of chemotherapy after primary surgical treatment (modified radical mastectomy). The median duration of tamoxifen use was 11 months (IQR 6–18 months). The socio-demographic and tumor characteristics are displayed in Table 1.

*SNP* genotyping results for *CYP2D6, CYP2C9, CYP2C19, CYP3A5, POR, ABCB1* and *UGT2B15* are presented in Table 2. All the genotype frequencies were in Hardy-Weinberg Equilibrium (*p* > 0.05). A total of 20 (25.9%) patients had *CYP2D6* copy number of 3 or more. The allele frequencies of CYP2D6 *5 (gene deletion), and *1 or *2 gene duplication/multiplication were 4.3 and 14.8%, respectively. The frequencies of *CYP2D6* activity score (AS) and phenotype categories are presented in Table 3. Based on the CPIC *CYP2D6* phenotype assignment, 61.7 and 22.2% of the patients are predicted to be normal metabolizers (NMs) and ultra-rapid metabolizers (UMs), respectively. The prevalence of poor (PMs) and intermediate metabolizers (IMs) was low, accounting for 1.2 and 3.7%, respectively.

### 2.2. Plasma Tamoxifen and Its Metabolite Concentration Profile

The plasma concentrations of tamoxifen and its metabolites are depicted in Table 3. The median concentration of endoxifen (7.94 ng/mL), was 2.6 times higher than 4-HT (3.04 ng/mL). The proportion of patients with low endoxifen concentration (<5.9 ng/mL) was 35.8%. This low level of endoxifen was detected in patients classified as PM/PM (1/1), IM/PM (3/3), IM/IM (1/1), NM/PM (6/9, 66.7%), NM/IM (5/12, 41.7%), and NM/NM (11/35, 31.4%) diplotypes. There was pronounced interindividual variability in endoxifen concentration (coefficient of variation 74.7%) and in metabolic ratio of endoxifen to NDM (coefficient of variation 59%) (Table 3).

**Figure 1:**
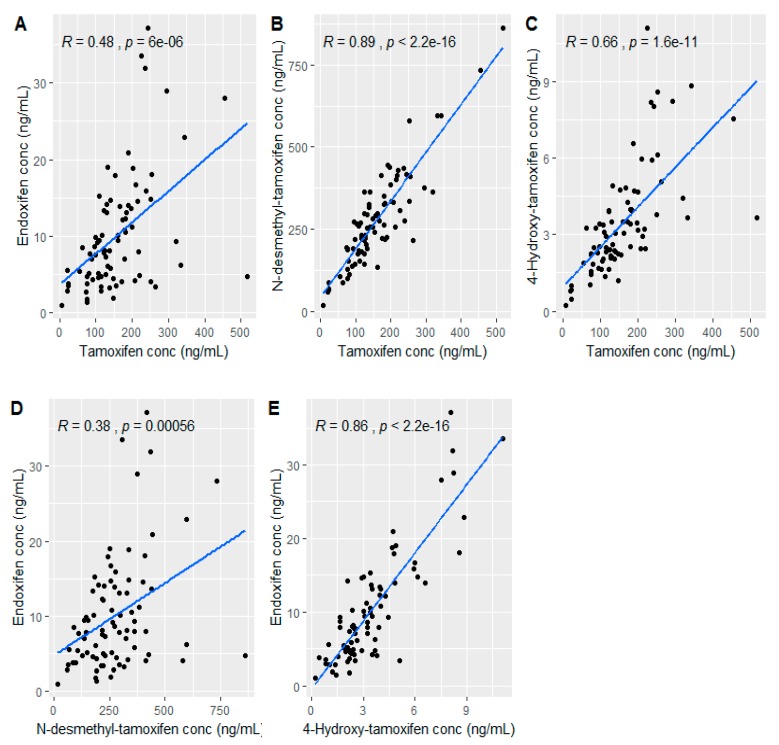
Scatter plot showing the Pearson’s correlation between endoxifen versus tamoxifen (**A**), N-desmethyl-tamoxifen versus tamoxifen (**B**) and 4-hydroxy-tamoxifen versus tamoxifen (**C**), endoxifen versus N-desmethyl-tamoxifen (**D**) and endoxifen versus 4-hydroxy-tamoxifen (**E**).

Pearson correlation analysis between tamoxifen and its metabolites revealed a strong positive association between tamoxifen and formation of NDM (Pearson correlation coefficient (*R*) = 0.89), and tamoxifen and formation of 4-HT (*R* = 0.66) (Figure 1). Thus, an increase in the concentration of tamoxifen was associated with a corresponding increase in NDM and 4HT levels. Formation of endoxifen is also strongly correlated with 4-HT level (*R* = 0.86). However, the correlation between NDM and formation of endoxifen (*R* = 0.38) as well as tamoxifen and formation of endoxifen (*R* = 0.48) was moderate.

### 3.3. Association of Pharmacogenetics and Pharmacokinetics of Tamoxifen Its Metabolites 

**Figure 2:**
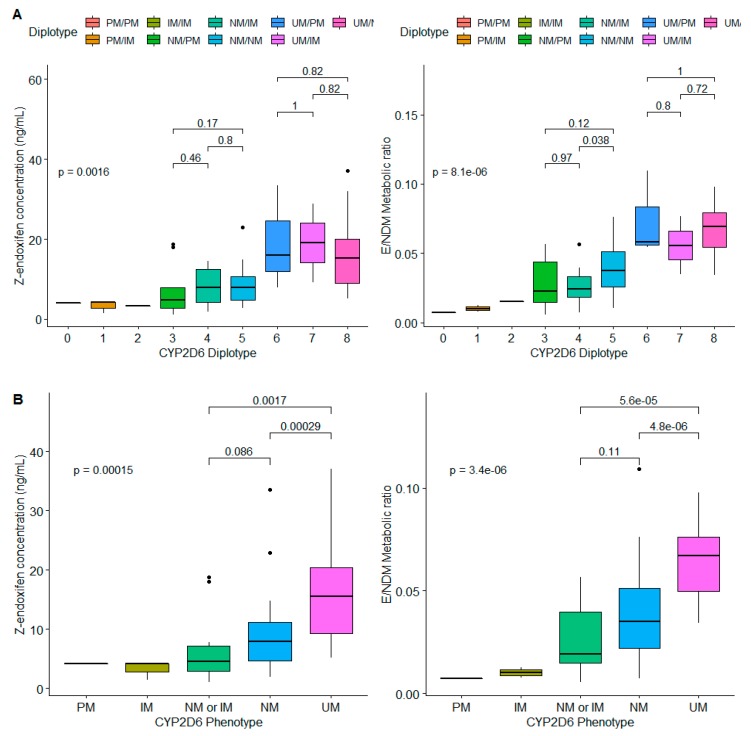
Plasma concentration of endoxifen and metabolic ratio (MR_E/NDM_) by *CYP2D6* diplotype (**A**) and phenotype (**B**).

Analysis of variance showed that the median endoxifen level, MR_E/NDM_ and MR_4HT/Tam_ were significantly different among *CYP2D6* diplotype (*p* < 0.001) or genotype-predicted phenotype groups (*p* < 0.001) (Figure 2). Patients with UM/EM, UM/IM and UM/PM diplotypes were associated with higher median endoxifen concentrations or metabolic ratios (MR_E/NDM_ and MR_4-HT/Tam_) (Figure 2A). Similarly, a corresponding increase in absolute endoxifen concentration and metabolic ratio (MR_E/NDM_) was observed with an increase in the activity score of *CYP2D6* phenotype (*p* < 0.001) (Figure 2B).

In patients categorized into *CYP2D6* NM/IM diplotype group, significant difference in MR_E/NDM_ (*p* = 0.036) and MR_E/4-HT_ (*p* = 0.022) was observed between genotypes *1/*10, *1/*17 and *2/*17. Among patients classified under NM/NM diplotypes, significant differences were also found between *1/*1, *1/*2, and *2/*2 genotypes in tamoxifen (*p* < 0.001), NDM (*p* < 0.001), and 4-HT concentrations (*p* < 0.001) as well as corresponding metabolic ratios; MR_E/NDM_ (*p* < 0.001), MR_E/4-HT_ (*p* = 0.025) and MR_4-HT/Tam_ (*p* = 0.007).

**Figure 3:**
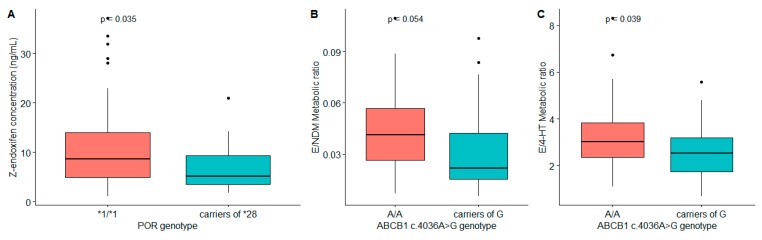
Plasma endoxifen concentration and metabolic ratios (MR_E/NDM_ and MR_E/4-HT_) plotted by *POR* (**A**) or *ABCB1 c.4036A>G* genotype (**B**,**C**).

In addition to *CYP2D6* genotype, a moderate association *POR* genotype with endoxifen level was observed (*p* = 0.035) (Figure 3A). On the other hand, *ABCB1* (rs3842) genotype showed a trend of association with MR_E/NDM_ (*p* = 0.054) and moderate association with MR_E/4-HT_ (*p* = 0.039). Carriers of the *G allele* for *ABCB1* (rs3842) had a lower MR_E/NDM_ (0.022 vs. 0.041; *p* = 0.054) and MR_E/4-HT_ (2.52 vs. 3.02, *p* = 0.039) ratios compared to the wild type genotype (Figure 3B,C).

Patients with at least one variant allele for *UGT2B15* (c.1568A>C, rs4148269) showed lower median plasma concentrations of tamoxifen (125.9 vs. 173.8 ng/mL, *p* = 0.045). The median NDM/Tam (1.86 vs. 1.54, *p* = 0.004) and E/4-HT (2.96 vs. 2.06, *p* = 0.029) ratios were also higher in carriers of *C* allele for *UGT2B15* (c.1568A>C, rs4148269) compared to the wild type genotype. However, there was no significant difference in median plasma tamoxifen metabolite concentrations or metabolic ratios between *CYP2C9*, CYP2C19 CYP3A5, UGT2B15*2 (c.253G>T) or *ABCB1 c.3435C>T* genotypes. No difference was also detected in the median plasma tamoxifen concentrations and its metabolites (*p* > 0.05), between pre- and postmenopausal patients, and different BMI groups as well as with age.

The impact of *CYP2D6* diplotype or phenotype on the interpatient variability of endoxifen or MR_E/NDM_ was modeled using linear regression. The results indicated that *CYP2D6* diplotype explained 28.2% of the variability in endoxifen concentration (coefficient of determination (*R^2^*) = 0.282, *p* < 0.001). Similarly, *CYP2D6* phenotype explained 26.3% of the variability in endoxifen concentration (*R^2^* = 0.263, *p* < 0.001). *POR* contributed 3.4% of the variability in absolute endoxifen concentration (*R^2^* = 0.034, *p* = 0.054). *CYP2D6* diplotype explained 44% of variability in MR_E/NDM_ and combined with *ABCB1c.4036A/*G genotype, it explained 46.7% (*R^2^* = 0.467, *p* < 0.001) of the variability in MR_E/NDM_. Phenotype and *ABCB1c.4036A>G* explained 40.9% of the variability in MR_E/NDM_ (*R^2^* = 0.409, *p* < 0.001). *ABCB1c.4036A>G* contributed 4% the variability in MR_E/NDM_ (*R^2^* = 0.040, *p* = 0.042).

The impact of *CYP2D6* on endoxifen variability and MR_E/NDM_ (a marker for *CYP2D6* metabolic activity) was assessed stratifying patients by *ABCB1* genotype groups. Interestingly, *CYP2D6* diplotype explained 43% of the variability in endoxifen concentration among *ABCB1c.4036G* carriers (*R^2^* = 0.43, *p* = 0.004) (explanatory power increased from 28.2 to 43%), compared to *A/A* genotype (*R^2^* = 0.231, *p* < 0.001). Similarly, *CYP2D6* diplotype explained 65.2% of the variability in MR_E/NDM_ (improved from 44 to 65.2%) among *ABCB1c.4036G* carriers (*R^2^* = 0.652, *p* < 0.001), compared to *A/A* genotype (*R^2^* = 0.356, *p* < 0.001).

## 3. Discussion

In the present study, we investigated the pharmacokinetic profile of tamoxifen and its active metabolites in breast cancer patients, and explored the impact of *CYP2D6*, *CYP2C9*, *CYP2C19*, *CYP3A5*, *POR, ABCB1* and *UGT2B15* genotypes on endoxifen and MR_E/NDM_ in Ethiopians, a population with high frequency distribution of active *CYP2D6* gene duplication or multiplication. Our major findings include; (i) a wide between-patient variability in plasma concentrations of tamoxifen and its metabolites, (ii) significant proportion of patients (35.8%) with subtherapeutic plasma endoxifen concentrations (<5.9 ng/mL), (iii) *CYP2D6* diplotype is a significant predictor of both endoxifen concentration and MR_E/NDM_, (iv) and a moderate effect of POR*28 on plasma endoxifen concentration and *ABCB1c.4036A>G* genotype on MR_E/NDM_. To our knowledge, this is the first study to investigate the pharmacokinetics and pharmacogenetics of tamoxifen in breast cancer patients from subSaharan African population and to explore the impact of *POR* and *ABCB1c.4036A>G* pharmacogenetics on tamoxifen PK.

Although *CYP2D6* is involved in various tamoxifen metabolic pathways, it is the sole enzyme responsible for the formation of endoxifen from NDM [1]. Previous studies have consistently demonstrated a significant gene-dose effect of *CYP2D6* polymorphism for tamoxifen metabolism [3,6,7,8]. Our study also showed an increase in endoxifen concentration and MR*_E_*_/NDM_ with increase in *CYP2D6* activity score, indicating a gene-dose effect of endoxifen formation. However, *CYP2D6* gene is highly variable among different ethnic groups, resulting in variations in concentrations of tamoxifen and its metabolites, mainly endoxifen [26,27].

In the present study, *CYP2D6* diplotype and *ABCB1c.4036A/G* genotype explained 46.7% the variability in MR_E/NDM_. With slightly different genotype interpretation applied for *CYP2D6* AS, previous study demonstrated that the contribution of *CYP2D6* to the IIV of endoxifen formation via NDM, as revealed from MR_E/NDM_, was 46% (Asians), 55% (Lebansese) and 55% (Caucasians) [3]. Our result is comparable to Asians but lower than Lebanese or Caucasians. The explained variability of absolute endoxifen concentration by *CYP2D6* diplotype in our study (28.2%) was also lower than that reported in Asians and Caucasians (39–58%) [7], suggesting that other factors could contribute for the bioavailability of endoxifen in our study population.

Previous studies reported that variations in the concentration of endoxifen can be related to variations in tamoxifen concentrations itself [28,29]. A high between-patient variations in plasma tamoxifen (CV = 56.2%) and NDM concentrations (CV = 53.1%) was also observed in our study. The Pearson’s correlation coefficient between endoxifen and tamoxifen (*R* = 0.48) as well as endoxifen and NDM (R = 0.38) also showed that, endoxifen level would only moderately be predicted by plasma levels of tamoxifen and NDM. This could mean that the extent of absorption of tamoxifen from the gut or the quality of the formulation or food-drug interaction could also be potential factors influencing the bioavailability of tamoxifen thereby impacting the plasma level of endoxifen.

Our study also showed significant differences in MR_E/NDM_ between patients with genotype CYP2D6 *1/*10, *1/*17 and *2/*17, which belong to EM/IM dipotype. Similarly, differences were also observed in endoxifen level between *1/*1, *1/*2, and *2/*2 genotypes which are grouped under same NM/NM diplotype groups (Figure 2). In agreement with our finding, recent study demonstrated that the concentrations of tamoxifen, endoxifen 4-HT, and NDM were significantly different between genotypes classed under same NMs [8]. The range of activity between CYP2D6*1/*1, *1/*2, and *2/*2 genotypes could be explained by the differences in the level of expression of mRNA, associated with the presence or absence of enhancer SNP in the enhancer regions distant to the *CYP2D6* gene [30].

Previous studies showed that tamoxifen efficacy is dependent on attaining a certain threshold of endoxifen level. According to Madlensky et al., women in the upper quintiles of endoxifen concentration, but not of tamoxifen and other metabolite levels, had a lower recurrence rate compared to those in the lowest quintile. Moreover, endoxifen concentration >5.97 ng/mL was associated with a 30% lower risk of additional breast cancer events and proposed as the therapeutic threshold for endoxifen [10]. A similar endoxifen level (13 nM) was also noted to occupy 90 % of the estrogen receptor (ER) in vitro and 93% of *CYP2D6* PMs fell below this concentration [12]. Subsequent study also demonstrated that, low endoxifen concentration (<5.97 ng/mL) was associated with distant relapse free survival [3], indicating the significance of this therapeutic threshold. The results of our study showed that significant proportion of Ethiopian patients (35.8%) were unable to attain this proposed therapeutic threshold for endoxifen concentrations. This is in agreement with previous study reported in Sweden (30%) [29], but higher compared to that reported in the U.S. (20%) [10].

In our study, the low level of endoxifen was detected in patients classified to PM/PM, IM/PM, IM/IM, NM/PM, NM/IM, as well as NM/NM diplotypes. Poor/intermediate metabolizer genotype was identified as a predictors of low endoxifen concentration in earlier study [10]. Moreover, it was also reported that 99% of PMs can be predicted by *CYP2D6* null alleles in homozygous variant or heterozygous genotype [31]. Our result is in agreement with this notion, given that all the null or lower activity diplotypes (PM/PM, IM/PM, and IM/IM) were associated with low endoxifen level. In such patients, the clinical outcome of the cancer could be less favorable, though its association was not investigated in the present study. Thus, in line with the recent CPIC therapeutic recommendations, *CYP2D6* PMs in our study are recommended alternate hormonal therapy such as aromatase inhibitors (AIs) for post-menopausal women or AIs along with ovarian function suppression in premenopausal women. Escalation of tamoxifen dose from 20–40 mg/day can also be considered for PMs if there is contraindications to AIs [32].

In contrast, other studies demonstrated that not all PMs have endoxifen concentrations below the proposed threshold concentration (5.9 ng/mL) [10,33]. For example, it was observed that 24 % of the PMs were still able to generate therapeutic concentrations of endoxifen despite the lack of metabolic activity of *CYP2D6* [33]. This could partly be attributed to environmental factors. A previous study demonstrated that black Africans were associated with lower rates of *CYP2D6*-dependent drug metabolism compared to Caucasians of the same apparent genotype [34]. Moreover, among individuals of the same *CYP2D6* genotype, it was revealed that Ethiopians in living in Sweden had higher *CYP2D6* metabolic activity than Ethiopians in Ethiopia [34], implying the profound impact of environmental factors in *CYP2D6* catalyzed drug metabolism. The frequency of *CYP2D6* PM (1.2%) and *CYP2D6* duplication (26%) identified in this study is lower as compared to previous report [34].

In the present study, patients with NM/IM (41.7%) and NM/NM diplotypes (31.4%), all belonging to NM phenotypes had lower endoxifen concentrations (<5.9 ng/mL). Recently, it was reported that the activity of *CYP2D6* is impacted not only by genetic variations within the *CYP2D6* gene, but also by regulatory mechanisms including genetic polymorphisms in the enhancer regions or differential expression of transcription factors, which can modulate the rate of translation of mRNA into protein [35]. Genotype panels typically interrogate a limited number of SNPs identifying more commonly observed *CYP2D6* alleles and provide limited information on gene copy number and structural variants [35]. It is therefore possible that a patient carries allele(s) that was not tested for and that may contribute to the variability seen in these groups. In addition, the impact environmental factors on *CYP2D6* catalyzed drug metabolism cannot also be underestimated [34]. Moreover, other individual-specific and biologic factors may also modulate *CYP2D6* expression levels or enzyme activity and contribute to the variability *CYP2D6* enzymatic activity. Emerging evidence showed the contribution of the microbiome on overall health and its role in drug metabolism and response [36]. A recent study also provided evidence that nutritional status, specifically fasting, alters P450-mediated drug metabolism in animal studies [37]. However, the extent a person’s microbiome, nutritional and fasting status influences CYP activity and *CYP2D6* metabolizer status, intra- and interindividual variability, in particular, remains to be investigated in future studies. Notably, the low level of endoxifen detected in large proportion of patients with predicted NM phenotypes in our study may suggest the need for therapeutic drug monitoring (TDM) of endoxifen in addition to genotyping for the common functional *CYP2D6* variant alleles.

Cytochrome P450 oxidoreductase (*POR*) is the only electron donor for all microsomal cytochrome P450 monooxygenases (CYP) responsible for oxidation of more than 80% of drugs [38]. In our study, we observed that POR*28 carriers were associated with low plasma endoxifen level, although the effect is moderate (*p* = 0.035). Previous reports indicated that genetic variants of *POR* (e.g., POR*28 (A503V)) were implicated in the impaired activity of *CYP2D6* and *CYP3A4* [39,40], possibly contributing to genetic variability in drug metabolism.

Apart from tamoxifen activating enzymes, genetic variants in drug transporters have been reported to influence pharmacokinetics and play a role to a variable degree in the clinical outcome of tamoxifen treatment and to confer treatment resistance for many anticancer drugs [41]. Results of the present study did not show significant effect of *ABCB1c.3435C>T* on tamoxifen and its metabolites level. However, it was observed that carriers of *G* allele for *ABCB1c.4036A>G* had a marginally lower MR_E/NDM_ (0.025 vs. 0.036; *p* = 0.054) compared to the wild type allele (*ABCB1*, *allele A*) indicating that polymorphism of *ABCB1 ABCB1c.4036A>G* could influence exposure to endoxifen. Interestingly, among *ABCB1c.4036G* carriers, improved predictive effect of the variability in endoxifen concentration (43%) and endoxifen formation from NDM (65.2%) was observed by *CYP2D6* diplotype. A previous study demonstrated that *ABCB1* influences the systemic exposure of tamoxifen and its active metabolites, 4-HT and endoxifen [16]. Moreover, several other studies also substantiated the impact of *ABCB1* in relation to clinical outcome of tamoxifen. *ABCB1* polymorphism was associated with risk of recurrence or disease free survival (DFS), in breast cancer patients on tamoxifen treatment [16,42,43].

Variant alleles of *CYP2C9*, *CYP2C19* and *CYP3A5* could alter tamoxifen metabolism and exposure to its metabolites especially formation of 4-hydroxy-tamoxifen [1]. However, no evidence of association was observed between *CYP2C9*, *CYP2C19* and *CYP3A5* genotypes and plasma concentrations of tamoxifen or its metabolites. Our finding is substantiated with previous studies [6,11,33]. On the other hand, other studies have reported lower plasma concentrations of 4-HT and endoxifen among carriers of CYP2C9*2 or *3 alleles [3,12]. The disparity in the results could be attributed to the difference in sample size or the variation in allele distributions.

In addition of the significant effect of *CYP2D6* genotype, variations in glucuronidation activity of the *uridine 5’-diphosphate-glucuronosyltransferase (UGT)* also seem to play a role in maintaining optimal levels of both 4-hydroxy-tamoxifen and endoxifen. Modified glucouronidation could affect half-lives of circulating tamoxifen and its metabolites thereby influencing the effectiveness of active metabolites in the treatment of breast cancer [44]. In the present study, patients with *C* allele for *UGT2B15*c.1568A>C (rs4148269) showed a lower plasma concentrations of tamoxifen, compared to those with wild type genotype. Although we could not determine plasma levels of tamoxifen-glucouronide, our finding may suggest increased glucouronidation of tamoxifen. Higher glucouronidation activity of *UGT2B15*c.1568A>C (rs4148269) genotype was also reported against endoxifen and 4-hydroxytamoxifen [44] and against oxazepam [45].

On the other hand, our result did not show significant variations in tamoxifen and its metabolites concentration with UGT2B15*2 (c.253G>T, rs1902023). Similarly, no correlation was detected between this polymorphism and both hydroxylated tamoxifen metabolites and their corresponding glucouronide product [12,44]. However, UGT2B15*2 (c.253G>T, rs1902023) was demonstrated to be a determinant of glucouronidation of oxazepam [45,46] and lorazepam [47] in the human liver, suggesting a substrate-dependent effect of this polymorphism.

Our study has some limitations. We present a significant impact of *CYP2D6* and a marginal effect of *POR* and *ABCB1* on the pharmacokinetics of tamoxifen and its metabolites. However, the sample size included in our study was not powered enough and might be a reason for lack of significant association of *POR* and *ABCB1* genotypes with tamoxifen and its metabolites PK. In addition, association of plasma concentration of the active tamoxifen metabolites to clinical outcome was not assessed. The treatment of cancer in patients with comorbidities can be challenging as these individuals are underrepresented in clinical studies [48]. In addition to the *CYP2D6* genetics that much of the controversy around tamoxifen therapy has focused, drug-drug interactions (DDI) involving *CYP2D6* in polypharmacy for co-morbidities markedly increases the complexity of tamoxifen metabolism and efficacy. The concurrent use of *CYP2D6* inhibitors and tamoxifen would be expected to substantially reduce endoxifen concentrations, and there is clinical evidence to support this expectation [6,9,49]. For example, NMs who were also taking *CYP2D6* inhibitors had endoxifen plasma concentrations that were 58% lower than NMs not on *CYP2D6* inhibitors. Similarly, IMs on *CYP2D6* inhibitors had endoxifen concentrations that were 38% lower than IMs not on *CYP2D6* inhibitors [6]. As tamoxifen has complex pharmacokinetics, with more than a dozen drug-metabolizing enzymes and transporters involved in its disposition, enzyme inducers may increase the activity of several of these pathways, including phase II enzymes, ABC transporters, and various CYP enzymes other than *CYP2D6* [50]. There is a growing evidence that enzyme inducers can substantially alter the disposition of endoxifen, reducing tamoxifen efficacy [51].

## 4. Materials and Methods 

### 4.1. Patients

This study was conducted at the radiotherapy center of Tikur Anbessa specialized hospital, Addis Ababa University, Addis Ababa, Ethiopia. A total of 81 female breast cancer patients who were on adjuvant tamoxifen were enrolled. These patients had completed a full course of chemotherapy and were on tamoxifen 20 mg/day for at least three months. None of the study participants were taking any known *CYP2D6* enzyme inhibitor as co-medication. The study protocol was approved by the Institutional Review Board (IRB) of the College of Health Sciences, Addis Ababa University (AAU) (Ref No: 011/16/2016), the Armauer Hansen Research Institute Ethical Review Committee (AAERC) (Ref No: PO26/16) and the Ethiopian National Research Ethics Review Committee (NRERC) (Ref No: 3.10/235/2017). Signed informed consent was obtained from individual study participants prior to study enrolment. Patients’ demographic, clinical and tumor characteristics including age, menopausal status, performance status, weight, height, body mass index (BMI), histologic type of the tumor, degree of differentiation, tumor size, lymph node involvement, hormone receptor status and co-morbidity status were collected.

### 4.2. Genotyping of CYP2D6, CYP2C9, CYP2C19, CYP3A5, POR, ABCB1 and UGT2B15

Whole blood sample was collected in EDTA containing vacutainer tube and genomic DNA was isolated from peripheral leukocytes using QIAamp DNA Midi Kit (Qiagen GmbH, Hilden, Germany) following the manufacturer’s instruction. SNP genotyping was performed using TaqMan^TM^ drug metabolism genotyping assay reagents (Applied Biosystems, USA) for allelic discrimination as described previously [52] with the following ID numbers for each SNP: C_27102425_10 for CYP2D6*2 (rs16947), C_27102431_D0 for CYP2D6*4 (rs3892097), C_11484460_40 for CYP2D6*10 (rs1065852), C_2222771_A0 for CYP2D6*17 (rs28371706), C_26201809_30 for CYP3A5*3 (rs776746), C_25625805_10 for CYP2C9*2 (rs1799853), C_27104892_10 for CYP2C9*3 (rs1057910), C_25986767_70 for CYP2C19*2 (rs4244285), C_27861809_10 for CYP2C19*3 (rs4986893), C_8890131_30 for POR*28 (rs1057868) and C_11711730_20 for *ABCB1c.4036A>G* (rs3842), C_7586657_20 for *ABCB1c*.3435C>T (rs1045642), C_27028164_10 for UGT2B15*2 (rs1902023), C_9440184_20 for UGT2B15*4 (rs4148269). The genomic DNA samples were amplified in 96-well plates on QuantStudio^TM^ 12K Flex Real-Time PCR system (Applied Biosystems, Life Technologies Holding, Singapore). The final volume for each reaction was 10 μL, consisting of TaqMan^TM^ fast advanced master mix (Applied Biosystems, Waltham, MA, USA), TaqMan^TM^ 20× drug metabolism genotyping assays mix (Applied Biosystems) and genomic DNA. The PCR conditions consisted of an initial step at 60 °C for 30 s, hold stage at 95 °C for 10 min and PCR stage for 40 cycles step 1 with 95 °C for 15 and step 2 with 60 °C for 1 min and after read stage with 60 °C for 30 s. The characterized SNPs were selected on the basis of their potential to influence the functionality of enzymes to affect the disposition and transport of tamoxifen or its metabolite.

### 4.3. Copy Number Variation

The *CYP2D6* gene copy number for the patient DNA samples was determined by TaqMan^TM^ Copy Number Assay (Life Technologies, California, USA) according to the manufacturer’s recommendations. The genomic DNA samples were amplified in 96-well plates on QuantStudio^TM^ 12K Flex Real-Time PCR system (Applied Biosystems, Life Technologies Holding, Singapore) using comparative ΔΔC_T_ method. *CYP2D6* exon 9 (TaqMan™ Copy Number Assay ID: Hs00010001_cn), and intron 6 (TaqMan™ Copy Number Assay ID: Hs04502391_cn) were used as primers. RNase P (ID: 431683) was used as the internal control for copy number analysis (Life Technologies). All the samples were run in triplicate. The final volume for each reaction was 10 μL, consisting of TaqMan™ Genotyping Master Mix (Applied Biosystems), TaqMan™ Copy Number Assay mix (20×, Applied Biosystems), TaqMan™ Copy Number Reference Assay mix (20×, Applied Biosystems). The PCR conditions consisted of an initial step at 60 °C for 30 s, hold stage at 95 °C for 10 min and PCR stage for 40 cycles step 1 with 95 °C for 15 and step 2 with 60 °C for 1 min and after read stage with 60 °C for 30 s. Relative quantification of *CYP2D6* gene copy number was performed using CopyCaller™ Software (Life Technologies). Genomic DNA samples with known *CYP2D6* gene copy number (0, 1, 2, 3 and 4 *CYP2D6* gene copies) obtained from a previous study [15] were used as controls.

### 4.4. Quantification of Plasma Tamoxifen and Its Metabolites

Blood samples were collected in EDTA tube, centrifuged at 2500 *g* for 10 minutes, and the separated plasma was stored at −80 °C until analysis. Tamoxifen (Tam) and its major metabolites N-desmethyltamoxifen (NDM), (*Z*)-4-hydroxytamoxifen (4-HT), and (*Z*)-endoxifen (*E*) were quantified in the TDM Laboratory, Department of Clinical Pharmacology, Karolinska Institutet (Stockholm, Sweden), by ultra-high-performance liquid chromatography (UHPLC) followed by electrospray tandem mass spectrometry (LC-MS/MS). Briefly, 400 µL internal standard solution (containing 2 ng/mL of *Z/E*-endoxifen-d_5_, and 4-OH-tamoxifen-d_5_, and 20 ng/mL of N-desmethyl-tamoxifen-d_5_, and tamoxifen-d_5_) was added to 200 µL of thawed plasma samples and mixed for 10 minutes in a micro plate shaker (Vx-2400 Multitube mixer, Troemner, Philadelphia, USA), followed by centrifugation at 3400 rpm at 4 °C for 10 min. The supernatant was then transferred to new glass vial and evaporated to dryness using vacuum centrifuge. The residue was dissolved in 50 µL methanol over a microplate shaker for 10 min and the resulting solution was then centrifuged at 3400 rpm for 10 min at 4 °C. Ten microliter (10 µL) of the solution was injected into the LC system (Dionex Ultimate 3000, UHPLC focused, Thermo Scientific, California, USA) coupled with a mass spectrometer (TSQ Quantiva, Thermo Scientific). Separation occurred on an Accucore C-18 column (150 × 2.1 mm, Thermo Scientific). The mobile phase for gradient elution consisted of solution A (10 mmol/L ammonium formate with 0.005% formic acid in water) and solution B (10 mmol/L ammonium formate with 0.005% formic acid in 99% methanol). The LC conditions were: column oven temperature of 40 °C, flow rate of 0.50 mL/min, autosampler temperature of 10 °C. Detection was in positive ion mode using electrospray ionization and monitored using Trace Finder software (Thermo Scientific). Under this condition, the retention times were 3.25, 3.26, 3.38, 4.03, and 4.11 min for E-endoxifen, Z-endoxifen, 4-OH-tamoxifen, N-desmethyltamoxifen, and tamoxifen, respectively. Calibration curves were constructed by plotting the ratio of the area of the compound and the internal standard against the standard analyte concentration. Three quality control (QC) samples involving, (i) low-level QC [QCL] (0.3 ng/mL of E and 4-HT and 3 ng/mL of NDM and Tam, (ii) mid-level QC [QCM] (8 ng/mL of E and 4-HT and 80 ng/mL of NDM and Tam), and (iii) high level QC [QCH] (80 ng/mL of E and 4-HT and 800 ng/mL of NDM and Tam) were also included at each run.

### 4.5. Genotype Based Phenotype Assignment

Phenotype classification from genotype was based on the recent Clinical Pharmacogenetics Implementation Consortium (CPIC) guideline for *CYP2D6* and tamoxifen therapy [32]. Functional status of each allele was assigned an activity score (AS) value ranging from 0 to 1 [0 for no function (*4, *5), 0.5 for decreased function (*10, and *17) and 1.0 for normal function alleles (*1, and *2)]. The combination of any two alleles represents the patient’s diplotype. *CYP2D6* activity score (AS) was then determined as the sum of the values assigned to each allele of the diplotype. Accordingly, *CYP2D6* AS was translated into patient’s phenotype status as poor metabolizer, PM (AS = 0), intermediate metabolizer, IM (AS = 0.5), normal metabolizer, NM (AS = 1.5–2) and ultra-rapid metabolizer, UM (AS > 2). Patients with AS of 1 were classified as a *CYP2D6* normal metabolizer or intermediate metabolizers (NM or IM).

### 4.6. Statistical Analysis

Descriptive statistics were computed to explore the demographic characteristics, clinical profiles, genotype frequencies of participants. *Chi*-square test was used to compare the observed and expected genotype frequency according to Hardy–Weinberg equilibrium. The plasma concentrations of tamoxifen and its active metabolites were described as median with interquartile range (IQR). Interpatient variability (IIV) of plasma endoxifen and endoxifen/N-desmethyltamoxifen metabolic ratio (MR_E/NDM_), a marker for *CYP2D6* metabolic activity, was described by coefficient of variation (%COV). ANOVA or independent *t*-test was used to determine the association of log transformed concentrations of tamoxifen and its metabolites and MR_E/NDM_ across *CYP2D6* diplotypes, phenotypes, non *CYP2D6* genotypes (*CYP2C9*, *CYP2C19*, *CYP3A5*, *POR*, or *ABCB1c.4036A>G*), and other patient specific factors (i.e., age, BMI, menopausal status, etc). Pearson correlation coefficient was determined to assess the magnitude of association between tamoxifen and its metabolites. The impact of *CYP2D6* diplotype and phenotype to explain the absolute concentration of endoxifen or MR_E/NDM_ was assessed using linear regression. Adjusted coefficient of determination (adjusted R^2^) was used to define the proportion of the variance in endoxifen concentration and MR_E/NDM_ that could be predictable from *CYP2D6* diplotype and phenotype. The data were analyzed using SPSS for Windows (version 21.0, IBM Corporation, New York, USA). Graphs were prepared using *ggplot2* package in R (version 3.5.1) [53].

## 5. Conclusions

In conclusion, we report a high rate of low plasma endoxifen level and wide between-patient variability in plasma concentration of endoxifen, the main therapeutically active metabolites of tamoxifen. *CYP2D6* genotype and its activity score is a significant predictor of plasma endoxifen exposure. Tamoxifen therapy guided by *CYP2D6* genotyping in clinical practice may assist treatment decision-making for optimal patient benefit. Therapeutic drug monitoring is also recommended for those with normal metabolizer phenotypes. The marginal effect of *POR* and *ABCB1* on tamoxifen and its metabolites PK found in this study needs further investigation in a larger sample size. Despite the simplified AS system recommended by CPIC for genotype-phenotype translation, our study signals further refinement of the approach, probably considering other sources of variability such as *POR* and *ABCB1*. Future studies are also recommended to investigate the impact of the *CYP2D6* genetic variations on the clinical outcome of breast cancer in Ethiopian patients.

## Figures and Tables

**Table 1:** Socio-demographic and tumor characteristics of tamoxifen treated breast cancer patients at the radiotherapy center, Tikur Anbessa Specialized Hospital, Addis Ababa, Ethiopia.

Parameters		Value
Socio-demographics		
Age (years, mean ± SD *)		39 ± 8.5
BMI (kg/m^2^, mean ± SD)		24.6 (±3.86)
Chemotherapy regimen used		**N (%)**
FAC ^∝^		37 (45.7)
AC		11 (11.1)
AC - T		35 (43.2)
Duration of tamoxifen use (months, median + IQR ^♣^)		11 (6–18)
Menopausal status		**N (%)**
Premenopausal		56 (69.1)
Postmenopausal		25 (30.9)
**Tumor characteristics**		**N (%)**
Histologic type of tumor	Ductal	79 (97.5)
	Lobular	2 (2.5)
Degree of differentiation	Well differentiated	21 (25.9)
	Moderately differentiated	44(54.3)
	Poorly differentiated	16 (19.8)
Lymph node involvement	Negative	15 (18.5)
	Positive	66 (81.5)
Distant metastatic site	No known distant metastasis	10 (12.3)
	Bone, lymph node, or lung only	63 (77.8)
	Liver, CNS, lung + other organs	8 (9.9)

^♣^ IQR inter quartile range; * SD standard deviation; ^∝^ FAC (5-Flourouracil 500 mg/m^2^, Adriamycin [Doxorubicin] 50 mg/m^2^, and Cyclophosphamide 500 mg/m^2^), AC (Adriamycin 50 mg/m^2^ and Cyclophosphamide 600 mg/m^2^), and AC-T (four cycles of Adriamycin 60 mg/m^2^ and Cyclophosphamide 600 mg/m^2^ followed by another 4 cycles of Taxol 175 mg/m^2^).

**Table 2:** The genotype and allele frequency distribution of tamoxifen metabolizing enzymes’ and transporter genes.

Gene	Variant allele	Allele frequency (%)
*CYP2D6*	*2	33.3
	*4	4.9
	*5	4.3
	*10	1.9
	*17	10.5
	*1×N or *2×N ^†^	14.8
*CYP2C9*	*2	4.3
	*3	7.4
*CYP2C19*	*2	11.7
	*3	1.2
*CYP3A5*	*3	67
*POR*	*28	12.4
*ABCB1 c.4036A>G*	G	14.8
*ABCB1 c.3435C>T*	T	16.9
UGT2B15 *2	T	20.2
UGT2B15 *4	C	40.3

^†^ Number of *CYP2D6* gene copies.

**Table 3:** *CYP2D6* activity score and plasma concentrations of tamoxifen and its metabolite and respective metabolic ratios.

*CYP2D6* Activity Score	Phenotype Group ^†^	N (%)
>2	UM ^¥^	18 (22.2)
2	NM	37 (45.7)
1.5	NM	12 (14.8)
1	NM or IM	10 (12.4)
0.5	IM	3 (3.7)
0	PM	1 (1.2)
**Plasma concentrations**	**values**	**CV (%) ^‡^**
Tamoxifen (ng/mL, median + IQR *^#^*)	138 (105–200.5)	56.2
N-Desmethyl-tamoxifen (ng/mL, median + IQR)	257 (185.5–343)	53.1
4-Hydroxy-tamoxifen (ng/mL, median + IQR)	3.04 (2.15–4.0)	61
Z-Endoxifen (ng/mL, median + IQR)	7.94 (4.68–13.75)	74.7
**Metabolic ratio**	**values**	**CV (%) ^‡^**
MR^ε^_E/NDM_ (median + IQR)	0.038 (0.021–0.06)	59
MR^γ^_NDM/Tam_ (median + IQR)	1.81 (1.51–2.26)	28.6
MR^λ^_4-HT/Tam_ (median + IQR)	0.022 (0.016–0.029)	38.8
MR^♠^_E/4-HT_ (median + IQR)	2.85 (2.10–3.73)	43.9

^†^ Phenotype translated based on Clinical Pharmacogenetics Implementation Consortium (CPIC) guideline for *CYP2D6* and tamoxifen therapy; ^¥^ UM ultrarapid metabolizer; NM normal metabolizer; IM intermediate metabolizer; PM poor metabolizer; ^‡^ coefficient of variation; ^#^ IQR interquartile range; ^ε^ metabolic ratio of endoxifen to N-desmethyl-tamoxifen; ^γ^ metabolic ratio of N-desmethyl-tamoxifen to tamoxifen; ^λ^ metabolic ratio of 4-Hydroxy-tamoxifen to tamoxifen; ^♠^ metabolic ratio of endoxifen to 4-hydroxy-tamoxifen.
